# Adaptive Colour Contrast Coding in the Salamander Retina Efficiently Matches Natural Scene Statistics

**DOI:** 10.1371/journal.pone.0079163

**Published:** 2013-10-30

**Authors:** Genadiy Vasserman, Elad Schneidman, Ronen Segev

**Affiliations:** 1 Department of Life Sciences and the Zlotowski Center for Neuroscience, Ben-Gurion University of the Negev, Beer-Sheva, Israel; 2 Department of Neurobiology, Weizmann Institute of Science, Rehovot, Israel; Dalhousie University, Canada

## Abstract

The visual system continually adjusts its sensitivity to the statistical properties of the environment through an adaptation process that starts in the retina. Colour perception and processing is commonly thought to occur mainly in high visual areas, and indeed most evidence for chromatic colour contrast adaptation comes from cortical studies. We show that colour contrast adaptation starts in the retina where ganglion cells adjust their responses to the spectral properties of the environment. We demonstrate that the ganglion cells match their responses to red-blue stimulus combinations according to the relative contrast of each of the input channels by rotating their functional response properties in colour space. Using measurements of the chromatic statistics of natural environments, we show that the retina balances inputs from the two (red and blue) stimulated colour channels, as would be expected from theoretical optimal behaviour. Our results suggest that colour is encoded in the retina based on the efficient processing of spectral information that matches spectral combinations in natural scenes on the colour processing level.

## Introduction

One of the strongest visual perceptions we have is that of the different colours in the environment, from the greenish hue of the forest to the bright colours of ripe fruit. Due to the behavioural importance (and beauty) of spectral visual information, much effort has been devoted to understanding how the retina informs the brain about the different spectral properties of a visual scene. First, an array of different types of photoreceptors which contain light absorbing photopigments sample the visual spectrum. Then the photoreceptors outputs are recombined in the retinal circuit. According to the standard interpretation, colour coding by the retina is based on classes of colour opponent ganglion cells, which are made up of sums and differences of local photoreceptors responses [1]–[5].

The complex nature of retinal computation and adaptation to scene statistics is evidenced in the response properties of the retinal ganglion cells [6], the only cells in the retina that project axons to the brain. Ganglion cell firing rates are matched not only to the mean or median of light intensity [7], [8], but also to the range of intensity fluctuations around the mean [9]–[11]. This adaptation to the nature of the fluctuations around the mean light, or “contrast adaptation”, implements a gain control mechanism. At low contrast, it increases the ganglion cell firing rate response to stimuli and amplifies the signal-to-noise ratio of ganglion cell responses. At high contrast, it decreases the firing rate response to stimuli and prevents saturation of the ganglion cell response [12]. This adaptation mechanism is the result of retinal circuitry, since previous studies [13]–[15] have shown that photoreceptors do not adapt to light level contrast.

It has been shown that there are major differences between the rod and cone pathways adaptation to the mean light conditions [16]. Since the rods are more sensitive to light and regenerate more slowly than cones their adaptation takes more time. As to the contrast adaptation, there is no evidence for contrast gain control in rod-bipolar pathway. According to the study by Beaudoin et.al, rod-bipolar pathway shows little or no adaptation to dim light contrast modulation [17]. The contrast gain control in response of spiking ganglion cells in this conditions are the result of an intrinsic property of the ganglion cell [18].

Retinal adaptation to the statistical properties of visual stimuli has been studied by many groups [10], [13], [14], [19], [20], with the bulk of the work on achromatic stimuli. Similar to achromatic light level adaptation, the retina also adapts to the mean light level within a specific spectral window, which is the first order statistic of the colour stimulus. Another possible adaptive mechanism is chromatic contrast adaptation, in which the variance in different spectral windows affects retinal encoding. This type of mechanism could enhance the efficiency of colour signal transmission from the retina to the brain or emphasize parts of the visual input that are more relevant to the animal [4], [21].

Finally, It should be noted that there are multiple time scales that govern the retinal adaptation processes from hundreds of milliseconds for the cone light level adaptation, continue with tens of seconds for contrast adaptation and up to tens of minutes for the rods light level adaptation [22]. Clearly, each one of these adaptation processes can contribute to the adaptation to the chromatic properties of light. This can be done either by changing the response properties of the photoreceptors themselves or by changing the properties of the retinal circuitry[22].

## Materials and Methods

### Ethical standards

All experiments were approved by the Institutional Animal Care and Use Committee of Ben Gurion University of the Negev and the laws of the State of Israel.

### Monitor radiometric and photometric calibration

CRT computer monitor spectral output was measured using a Red Tide USB650 CCD Spectrometer (Ocean Optics). Spectrometer spectral output was calibrated to absolute radiometric units using the LS-1-CAL calibrated tungsten halogen lamp (Ocean Optics). All measurements were performed with a 1000-µm patch cord optical fiber (Ocean Optics).

### Electrophysiology

Experiments were performed on the adult tiger salamander (*Ambystoma tigrinum*). Retinas were isolated from the eye and peeled from the sclera together with pigment epithelium. Retinas were placed with the ganglion cell layer facing a multi-electrode array with 252 electrodes (Ayanda Biosystems) and superfused with oxygenated (95% O_2_, 5% CO_2_) Ringer medium at room temperature [23], [24]. The electrode diameter was 10 µm and electrode spacing varied from 40–80 µm. Extracellularly recorded signals were digitized at 10 kSamples/s on four personal computers and stored for off-line spike sorting and analysis.

### Visual Stimulation

**Figure 1:**
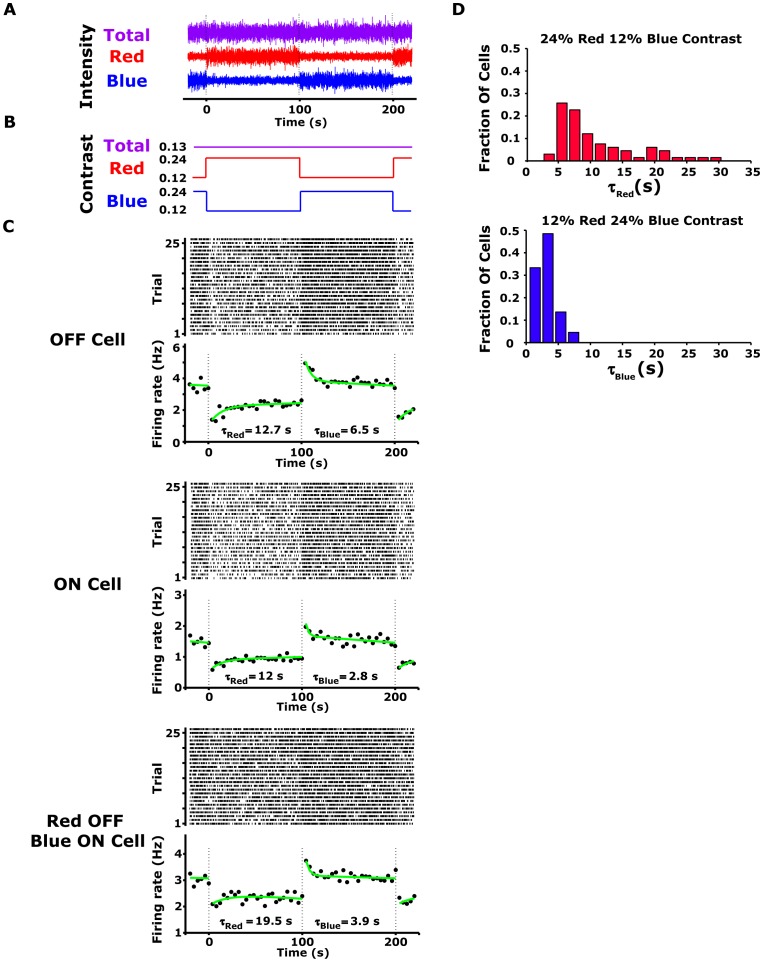
Spectral contrast adaptation in salamander retinal ganglion cells. (A) Sample of the red-blue stimulus used in the experiment; the mean intensity of each colour and the total light intensity were held constant (B) Schematic representation of the colour contrast modulation used in the experiment. Each 200-s period of the red-blue stimulus trial contained 100 s of random flicker at high red (24% contrast) and low blue contrast (12% contrast) followed by 100 s at low red and high blue contrast. Total light contrast remained constant throughout the experiment. (C) Raster plot and peri-stimulus time histogram (PSTH) in response to 52 contrast modulation cycles, calculated with 4-s bins for OFF (upper panel) and ON (lower panel) ganglion cells. The PSTH curves were fitted with exponential curves (green) with time constants in the range of 5–20 s. (D) Histogram of the time constant (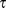) for OFF ganglion cells for high red contrast (upper panel) and high blue (lower panel) contrast modulation (n = 55).

The experiment comprised red and blue colour modulations generated by red and blue CRT monitor guns (ViewSonic, G90FB). The spectral curves of red and blue monitor guns have maximal light intensities close to the peak sensitivities of, respectively, tiger salamander long and short wavelength sensitive cones (Figure S1). Compared to the short wavelength sensitive cone, the UV cone, which has a peak sensitivity of 364 nm [25], reached about 5% stimulation in response to the blue monitor gun, and hence may be ignored. Spatially uniform red and blue light was projected onto the salamander retina from a CRT video monitor at a frame rate of 60 Hz using standard optics[26]. We encoded the visual stimulation using an 8-bit colour mode, i.e., each one of the colours had 256 levels of brightness. A new red or blue stimulus intensity was chosen every 33 ms from a Gaussian probability distribution with a mean intensity 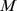 and a standard deviation 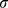 [13] (Figure 1A, 1B). Contrast was defined as 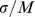. The contrast varied between different experiments and was specified for blue and red colours. The total mean intensity was kept constant at 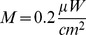 per channel at the screen which is equivalent to a total flux of 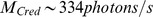 for the long wavelength sensitive cone, 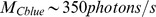 for the short wavelength sensitive cone and 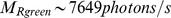 for the middle wavelength sensitive rod, 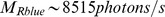 for the short wavelength sensitive rod. The contrast was 0.24 or 0.21 for high contrast flicker colour, and 0.12 or 0.14 for low contrast flicker colour.

### Analysis

To interpret ganglion cell responses to the flickering uniform spectral light, we used the linear-nonlinear (LN) cascade analysis described previously [10], [13], [14], [19], [20], [27]. The LN model predicts the transformation between light intensity and ganglion cell response by first linearly filtering the light intensity and then passing the filter output through a static nonlinearity. To fit an LN model to the cellular response, we need to estimate the linear filter and the nonlinearity. In our case, since the stimulus comprises two different channels, red and blue, we need to find the two filters that are associated with each channel and the two-dimensional nonlinearity that gives an estimate of the firing rate.

If stimulus intensity is drawn from a Gaussian distribution as in our case, the red and blue linear filters,
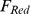
and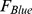, respectively, are proportional to the spike triggered average [19], [27]. This is: 
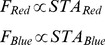
(1)where we need to set the proportionality constant. The 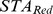 (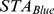) was defined as the average red (blue) stimulus preceding a spike in the cell, i.e., the sum of the stimuli 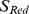 (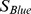) preceding each spike that occurred at time 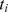
divided by the total number of spikes_n_ :



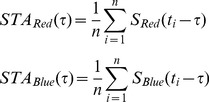
(2)To find the static nonlinearity, the red and blue stimulus components, 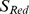 and 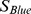, were convolved with the red and blue filters, respectively, by computing:
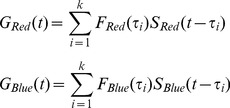
(3)where 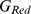 and 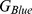
are the generator signals associated with each channel.

The fixed, two-dimensional nonlinearity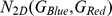 was then calculated by plotting the cell firing rate 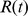 against 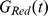 and 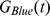 and averaging the values of 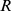
over bins of 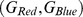 plane. Finally, LN model prediction was calculated as:
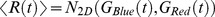
(4)


The LN model has an internal degree of freedom due to the ability to scale the generator signal 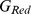 and 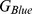 axes of the nonlinearity and the amplitude of the linear filters by the constant and its inverse, respectively, without changing model output. To solve this ambiguity, we adapted the calibration method used previously by Chichilnisky et al. [19], [27]. The basic idea was to superimpose the nonlinearities for each statistic by scaling 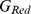 and 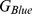. We scaled each linear filter 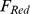
(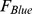), dividing it by filter amplitude and by input signal standard deviation 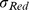
(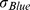), to correctly scale 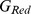 and 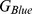. Scaling of the linear filter was insufficient to enable superimposing the nonlinearities because the scaling alone cannot account for the rotational modification. Thus, to superimpose the nonlinearities, we also rotated the nonlinearities by the angles 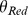 and 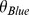 for high red and high blue contrast stimuli, respectively, setting each 2D nonlinearity parallel to the abscissa. The angles of rotation, 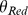 and 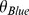 were found by fitting the nonlinearity with the sigmoid function 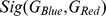:
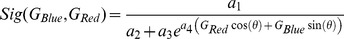
(5)where 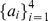
and the angle 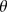 are free parameters.

After rotating the nonlinearities parallel to the 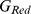 axes, we collapsed the two-dimensional nonlinearity into one dimension by averaging it along the 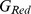 axes. That is, after the rotation, each of the two-dimensional nonlinearities 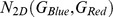 was replaced by the one-dimensional nonlinearity 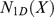, which is a function of 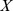:
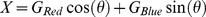
(6)


The simplified LN models that are described above are constructed based on the assumption that the stimulus has a Gaussian distribution. In the case of Non-Gaussian distribution of the stimuli, the assumption that the linear component of the model is proportional to the STA does not hold anymore. In this case we expanded the LN model into General Linear Non-Linear model (GLM). Similarly to LN model the GLM model is composed of linear filter and nonlinearity. The linear filter and nonlinearity parameters were estimated by using the maximum likelihood parameters estimation approach [28].

### Model Validation

**Figure 2:**
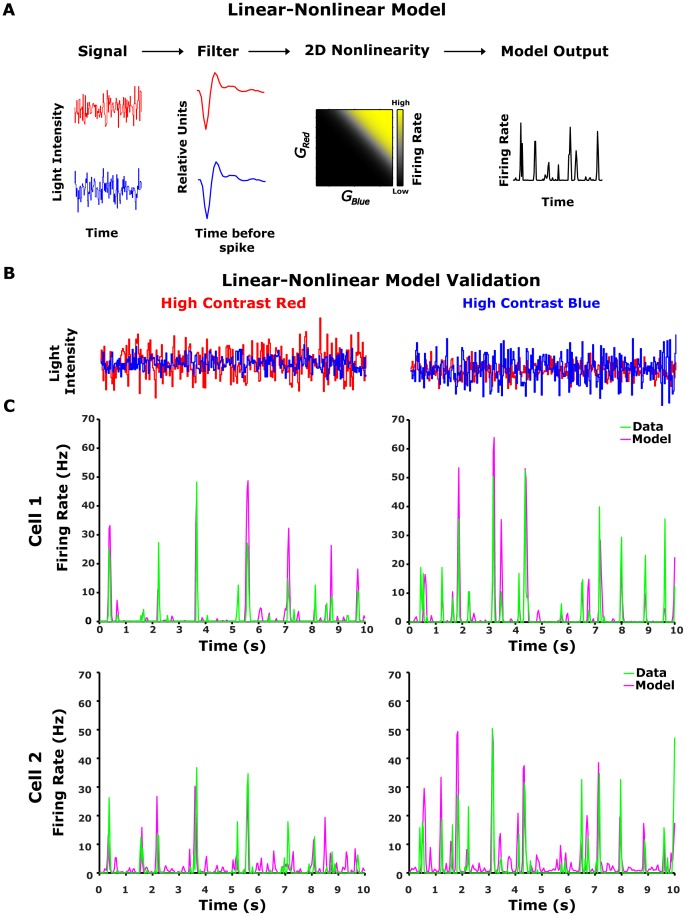
Linear-nonlinear model analysis. (A) Schematic overview of the LN model. The red and blue stimuli were convolved with red (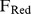) and blue (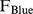) linear filters, respectively, and the product was passed through a static nonlinearity (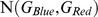), resulting in a prediction of the LN model for ganglion cell firing rate. (B-D) Testing the LN model for two colour contrast conditions. Left column: high red (24%) and low blue (12%) colour contrast. Right column: high blue (24%) and low red (12%) colour contrast. (B) Intensities of red and blue light stimuli (red and blue curves) used in the experiment. (C) LN model prediction for two example cells compared to the experimental data. Data was averaged over 20 trials (bin size 33 ms). The LN model output (magenta coloured line), constructed from a separate data set, follows the data (green line) closely (correlation coefficients were 0.72 and 0.75 for the Cell1 (upper panel) and 0.36 and 0.61 for Cell2 (lower panel) for high red and high blue contrasts respectively).

We tested the predictive ability of the LN model by building the model from one set of data and testing it on a second set of data (Figure 2C). The model comprised red and blue linear filters for each colour channel, followed by the rotation matrix 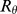 and the one-dimensional nonlinearity 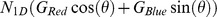 as described in the previous section. The LN model parameters for different statistics were fitted by presenting the ganglion cell model with 52 cycles of 200-s non-repeated segments (100 s of high contrast red and low contrast blue stimulus, followed by 100 s of high contrast blue and low contrast red). The model was fitted using the neural activity recorded 50 s after the contrast switch. To test the model, we used 20 repeats of 100 s of high contrast red and low contrast blue, followed by 100 s of high contrast blue and low contrast red. The data used for testing were taken from the last 50 s of each half-cycle, during the repeated stimulus. Noise in the measured response was reduced by averaging 20 repetitions of an identical light stimulus. The predictive power of the model was quantified by the correlation coefficient between the predicted and measured firing rates [14], [20], which were around 0.52 for both stimuli (Table 1).

**Table 1:** Population summary of the performance of different LN models of retinal response to colour stimuli.

			High Red Contrast		High Blue Contrast	
			Mean	Std. Deviation	Mean	Std. Deviation
**Model**	**LN**	Chromatic rotation	0.5201	0.1204	0.5439	0.104
		Achromatic summed	0.483	0.1218	0.5241	0.1021
		2D-fit	0.4993	0.1186	0.5271	0.0921
	**GLM**	Chromatic rotation	0.5653	0.115	0.5771	0.1253
		Achromatic summed	0.3417	0.2513	0.5153	0.1332

**Figure 3:**
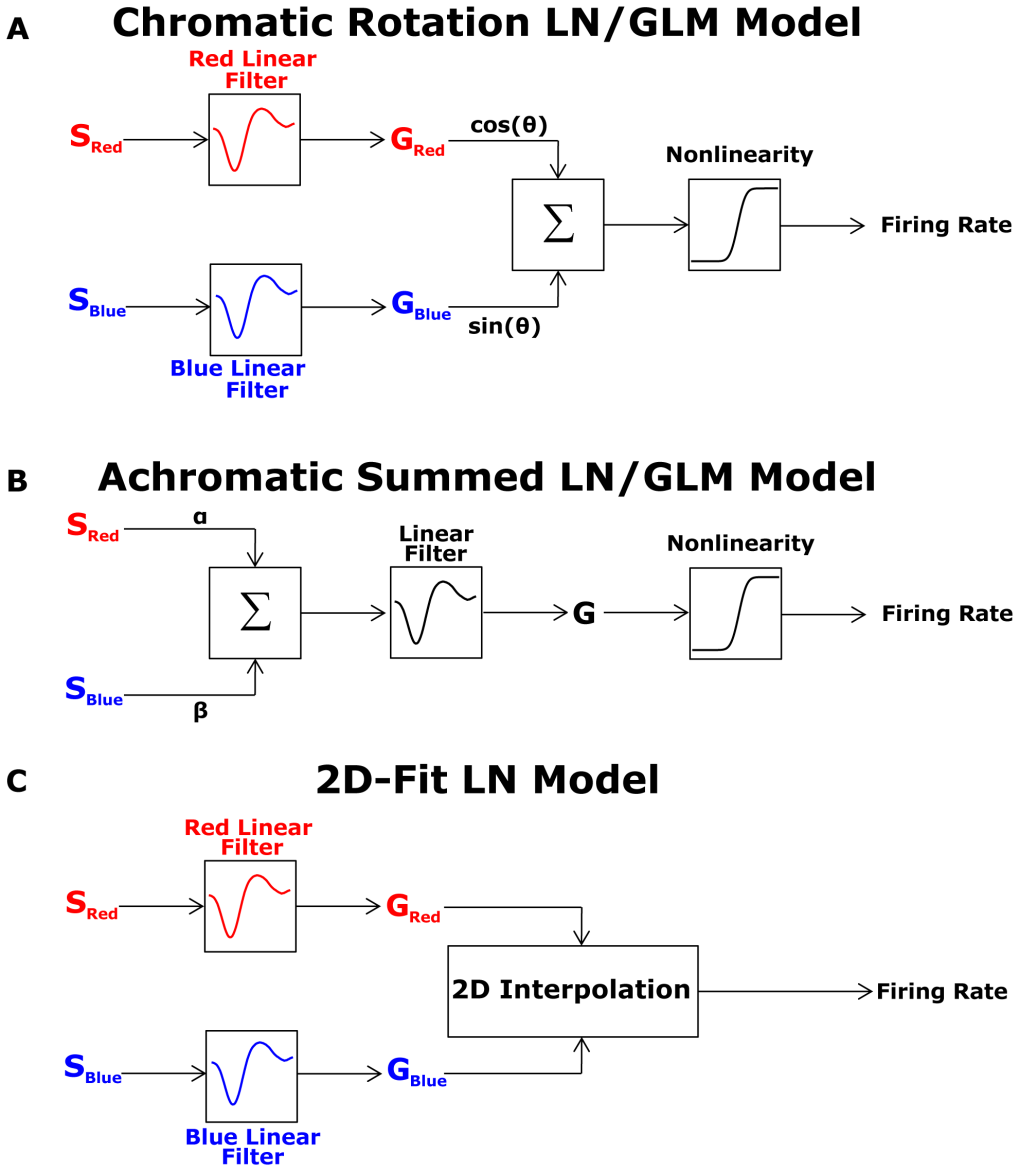
Summary of schematic structure of all tested Linear-nonlinear models. (A) Chromatic Rotation LN model. This is the main model we have presented in the main text. The two colour channels are filtered separately, than fed into a rotation which weight them and feed the result to a one-dimensional nonlinearity. (B) The Achromatic Summed LN model. The two channels are summed with two fixed weights that are optimized to get the best response in the training dataset, but are not allowed to adapt afterwards. Then the summed signaled is transferred to a one dimensional LN model with a single filter and non-linearity which is allowed to adapt. This is the simplest model and it has achromatic adaptation nature since it is blind to the two colour channels when the adaptation process takes place. (C) 2D-Fit LN model. The two colour channels are filtered separately and fed into the two dimensional nonlinearity. Since we do not observe all possible combinations of the generator signals we interpolate the two dimensional nonlinearity to obtain a smooth representation of the connection between generator signals and firing rates. This model represents the minimal possible assumptions.

The LN and GLM models that were tested are presented at Figure 3. The stimulus with high blue contrast was a combination of red input (12% contrast) and blue input (24% contrast); the high red contrast stimulus was a combination of red (24% contrast) and blue (12% contrast) – see Figure 1A. The models' performance was quantified for each ganglion cell by the correlation coefficient between the model's output and the average firing rate of the cell. Here we summarize accuracy of the different models for n = 64 ganglions cells, we found that the chromatic rotation LN model is superior over the achromatic summed and 2D-fit LN models.

### Natural scene colour statistics

The light spectrum was measured with a spectrally calibrated Red Tide USB650 CCD spectrometer, equipped with 1000 µm diameter patch cord optical fibre (both from Ocean Optics). An optical fibre was placed inside a black plastic tube at a distance of 50 mm from a 5-mm diameter pinhole. This *camera obscura* form of device was used to restrict the measured visual field angle to 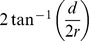 (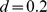
mm, average receptive field diameter of the adult salamander, 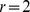
mm, average eye radius of the adult salamander), which is equivalent to the average visual scene angle projected on the salamander ganglion receptive field [24]. Natural scene spectral curves were measured at the Tel Dan Nature Reserve, Israel, (33.249° N, 35.652° E), which is a well-known natural habitat of the Israeli fire salamander (*Salamandra salamandra*) [29]. All measurements were taken in an area where the salamanders were highly abundant, over four days in mid-October 2008 between 8 AM and 4 PM when the reserve was open to the public.

### Transformation of broadband spectra light intensity to the tiger salamander cone space

Long and short wavelength sensitive cone responses, 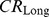 and 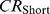 respectively, were calculated using Grassman's law[30]: 
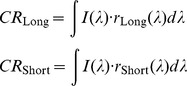
(7)where 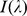 and 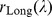 and 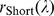
were broadband light intensity and long and short wavelength cone sensitivities, respectively, measured at wavelength 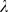. Cone spectral sensitivity data was adapted from Makino et al. [25]_ENREF_34_ENREF_33.

## Results

### Colour contrast adaptation in the salamander retina

To demonstrate and quantify the nature of colour contrast adaptation in the retina, we used multi-electrode recordings of the tiger salamander (*Ambystoma tigrinum*) retina responding to wide field coloured stimuli. The salamander retina has five classes of photoreceptors: two rods, two cones that respond mainly to visible light (a long wavelength sensitive cone with a peak sensitivity at 610 nm and a short wavelength sensitive cone with a peak sensitivity at 444 nm), and an ultraviolet (UV) sensitive cone (with a peak sensitivity at 364 nm) [25]. It is clear that salamanders use the spectral specific information coming from the retina, as they exhibit colour guided behaviour [31], [32]. The lack of cones with peak sensitivity in the green region of the visual spectrum and the insensitivity of the UV cone to human-visible light allowed us to explore the retinal response to combinations of the red and blue colour channels using a standard CRT monitor.

### Retinal ganglion cells adapt their firing rates to the spectral properties of visual stimuli

We recorded the activity of a total of 569 ganglion cells from six isolated retinas of six different animals under different stimulus conditions using a planar array with 252 micro-electrodes (Materials and Methods). Each retina was exposed to long uniform-field flicker stimuli comprising both blue and red inputs. In each frame (33 ms refresh rate), the light intensities of the red and the blue colour channels of the monitor were chosen independently from Gaussian distributions with the same mean intensities but with standard deviations of 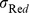 and 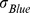, respectively. The total screen luminance as a function of time was then given by 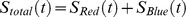.

To explore retinal adaptation to the spectral variance of the environment, we exposed the cells to long, continuous sequences of stimuli, and changed the contrast of each of the colour channels of the monitor every 100 s while keeping the mean intensity of each channel constant. The change in the two channels was abrupt (within one frame) and coordinated, such that the contrast of the summed input signal was invariant (Figure 1A, 1B), namely, 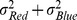 was kept constant. We thus presented the retina with stimuli drawn from one distribution and then switched to another, such that the two stimulus distributions had the same average light levels and the same total contrasts, but one stimulus set was dominated by the contrast of the red channel and the other by the contrast of the blue channel. Here, the contrast in each channel was defined as the ratio between the standard deviation and the mean of the intensity within that specific channel. If the retina adapts to colour contrasts, then there should be a corresponding change in retinal response properties when the stimuli are changed.

The responses of retinal ganglion cells to the changing red-blue contrast stimuli revealed that indeed, colour contrast adaptation begins in the retina. The firing rates of ON and OFF retinal ganglion cells were examined in response to stepwise changes in the contrasts of the red and blue channels while preserving their overall sum (Figure 1C). The firing rates of all cells increased in response to increases in the blue signal contrast, and decreased when the blue contrast was decreased (Figure 1C). The abrupt change in spike rate in response to the altered colour contrasts was followed by a slow, opposing change in the firing rate similar to the qualitative behaviour that was previously shown for achromatic contrast adaptation [10], [13]. As was observed for achromatic adaptation [10], we found that after a chromatic contrast step, the firing rate adapted exponentially (green curve, Figure 1C). The decay in firing rate observed after an increase in blue colour contrast was almost twice as fast as its recovery following a reduction in blue colour contrast (Figure 1D). Similar adaptation dynamics were found for both ON and OFF cell types.

We repeated the same experiment, but this time calibrated the stimulus according to the photoreceptors' absorption spectra and kept the same overall contrast in photometric units (Figure S2, 3). This set of stimuli therefore only took into account differences at the single photoreceptor level but not differences due to different numbers of photoreceptors of different types. We found that ganglion cells adapted to such photometrically normalized colour contrast modulation in a similar way to that observed with stimuli normalized to the physical units (Table 2).

**Table 2:** The dependence of the rotation angle on the statistics of stimulus.

	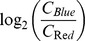	N	Mean	SD	SE	95% Confidence Interval for Mean	
						Lower Bound	Upper Bound
Radiometric	−1	106	52.13	3.98	.38	51.36	52.90
	−0.5	90	59.64	3.01	.31	59.01	60.27
	0	83	68.77	4.99	.54	67.68	69.87
	0.5	90	75.66	1.88	.19	75.26	76.05
	1	106	77.23	2.59	.25	76.73	77.73
Correlated	−1	42	55.13	3.31	.51	54.10	56.16
	−0.5	33	61.16	3.38	.59	59.95	62.36
	0	23	69.27	2.23	.46	68.30	70.24
	0.5	33	77.82	2.65	.46	76.88	78.76
	1	42	78.48	3.12	.48	77.51	79.45
Photometric	−1	35	54.98	4.04	.68	53.59	56.37
	−0.5	39	59.02	4.14	.66	57.68	60.37
	0	34	65.52	3.47	.59	64.31	66.73
	0.5	39	75.67	2.37	.38	74.90	76.44
	1	35	81.30	2.39	.40	80.48	82.12

We have performed three sets of experiments with different light statistics and estimated the average rotation angle 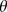, of the CR-LN model, over the population. The experimental conditions were: **A**. Light intensities were drawn from a Gaussian distribution (Radiometric units); this is the data set we have used in the main text. **B**. Light intensities were drawn from a Gaussian distribution with the same marginals for each channel as in **A**, but with correlation of 0.84 between the red and blue channels. This was done in order to simulate conditions similar to the ones found in natural scenes, where strong correlations between the different colour channels were observed (main text). **C**. The light intensities were calibrated according to the photoreceptors absorption spectra and kept the same overall contrast in photometric units. Then photometrically calibrated light spectra were drawn from Gaussian distribution as in A. The confidence intervals were calculated using Wilcoxon's test in SPSS. N, SD and SE stand for number of analyzed ganglion cells, standard deviation and standard error respectively.

### A Chromatic Rotation Linear-Nonlinear model captures ganglion cell colour encoding

To understand adaptive colour encoding by the retina, we constructed a chromatic version of the linear-nonlinear model of the ganglion cell responses to colour stimuli. Linear-nonlinear (LN) models have been used extensively to describe the responses of ganglion cells to various stimuli [13], [19], [20], [27]. In these models, the stimulus 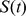 was passed through a linear filter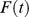, the output of which is given by the convolution of stimulus and filter: 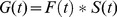
(8)where 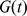 is the generator function. Finally, 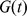 is passed through a nonlinear function N*(.)*, which determines the output firing rate of the neuron:



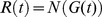
(9)The chromatic LN model we used here received multi-dimensional input where each dimension was given by one colour channel (Figure 2A). The red and blue inputs, 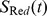 and 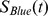, were each transformed by their respective filters, 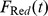 and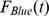, and these were fed into a non-linear function. In general, the firing rate of the cell 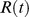 was given by a two dimensional (2D) nonlinear function 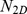 operating on the blue and red generator signals (Materials and Methods, Equation 4). We found, however, that proper weighting of the colour channels (see Materials and Methods), which captured the way the ganglion cells combine the colour contrast information, enabled us to build a successful chromatic LN model using only one dimensional (1D) nonlinearity. Thus, the firing rate of the cell could be approximated by a sigmoid function of the linear weighting of the two colour inputs:
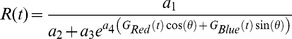
(10)where 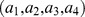 are the parameters of the 1D nonlinearity, and 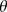 determines the relative weight of each colour channel, defining the direction of a sigmoid function (or nonlinearity front) in the plane of 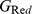 and 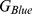, (see Materials and Methods, Equation 5). Recall that the LN model has an internal scaling degree of freedom: the linear filter and the corresponding input axis of the nonlinear function can be scaled by the same multiplicative factor without changing the output of the model (Equation 8 and 9) [19]. We therefore set the generator functions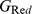 and 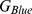, to have a unit variance (Materials and Methods, Analysis).

We analysed the performance of our LN model by constructing it from one set of data, and testing it on another (Materials and Methods, Figure 2C). The model's performance was evaluated by the correlation between predicted and measured firing rates for each ganglion cell. The prediction captured a significant part of the structure of the measured response, with a mean correlation coefficient of 0.52±0.12 and 0.54±0.1 (SD, n = 64) for high red and high blue contrast stimuli respectively (Table 1).

We have also constructed similar LN models for the photometric units stimulus and found similar behavior (Figure S3 and Table 2). In addition, we examined the LN model with correlated red-blue stimuli (Figure S4 and Figure S5) and found that the rotation angles were similar to that found with uncorrelated red-blue stimuli (Table 2). To conclude, the results hold under different stimulus conditions.

### Analysis of performance of alternative models

**Figure 4:**
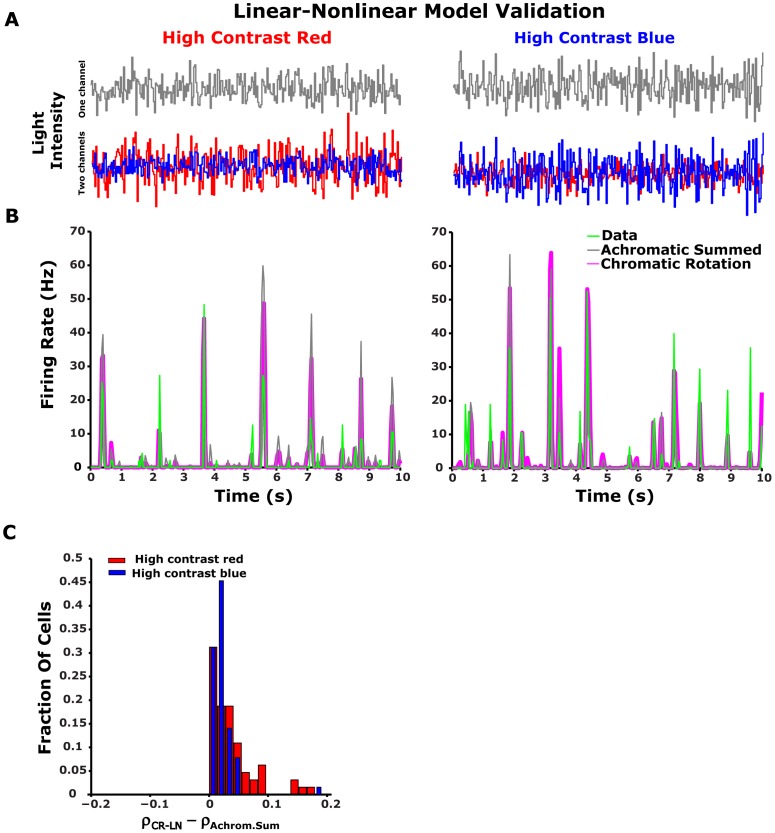
Performance of alternative Linear-nonlinear models. (A-B) Testing alternative LN models. Left column: high red (24%) and low blue (12%) colour contrast. Right column: high blue (24%) and low red (12%) colour contrast. (A) Intensities of red and blue light stimuli (red and blue curves) used in the experiment. Weighted sum of red blue stimuli was used as an input for the achromatic summed LN model (grey curve). (B) CR-LN model (Figure 3A) prediction compared to the output of the achromatic summed LN model (Figure 3B), to experimental data. Data was averaged over 20 trials (bin size 33 ms). The CR-LN model output (magenta coloured line), constructed from a separate data set, follows the data (solid line) closely, giving a better prediction than the achromatic summed LN model. (C) The histogram of the difference between the correlation coefficients for the CR-LN model and for the achromatic summed LN model for the analyzed ganglion cells (n = 64 cells). The positively skewed histogram suggests that the CR-LN LN model represents a better prediction than the achromatic summed LN model.

We compared the performance of several alternative LN models to the CR-LN model presented above (see Figure 3A). We first tried an achromatic summed LN model (Figure 3B) that was constructed by weighting and summing the input colour channels with some constant weights which were obtained by fitting model to data. The result was then fed to a 1D nonlinearity, as described previously [13], [19], [20], [27]. We found that the CR-LN model was superior to this achromatic summed LN model in accurately capturing the responses of the different ganglion cells (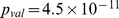
and 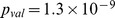 for high red and high blue contrasts respectively, Wilcoxon test, see Table 1 and Figure 4B, 4C).

**Figure 5:**
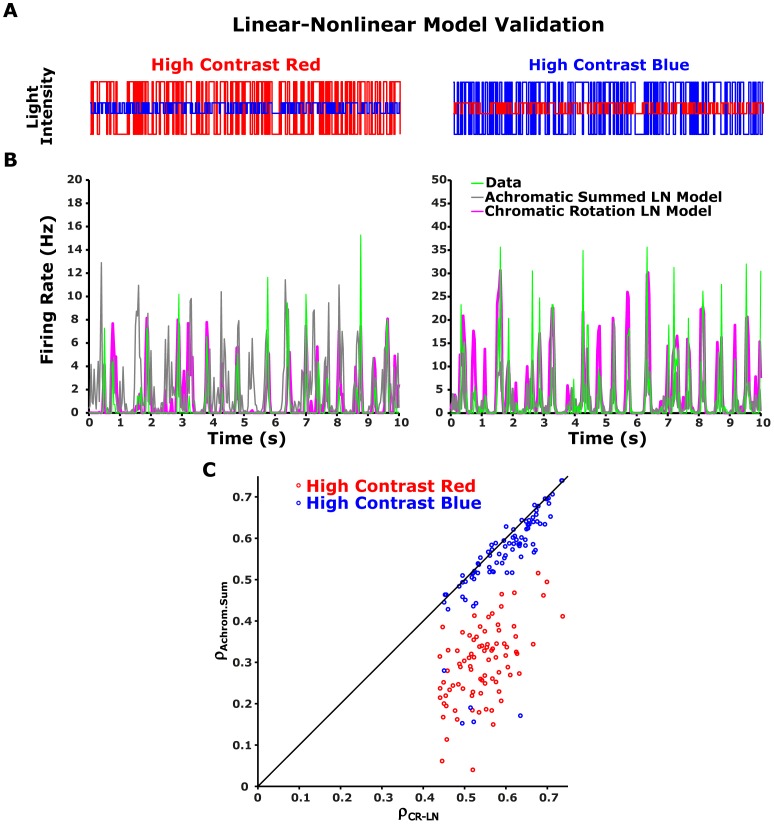
Performance of 2D-fit Linear-nonlinear model. Left column: high red (24%) and low blue (12%) colour contrast. Right column: high blue (24%) and low red (12%) colour contrast. (A) Intensities of red and blue light stimuli (red and blue curves) used in the experiment. (B) 2D-fit LN model (Figure 3D) prediction compared to the output of the chromatic rotation LN model (Figure 3A), and to experimental data. Data was averaged over 20 trials (bin size 33 ms). The 2D-fit LN model output (black line), constructed from a separate data set, follows the data (green line) closely, however the performance of the 2D-fit LN model was similar to the performance of the chromatic rotation LN model (Table 1).

We also compared the prediction of the CR-LN model using 60% and 12% high and low contrast stimuli (Figure 5). Since the normal distribution for high contrast stimuli was too broad to fit in the CRT monitor output range we used binary stimuli instead of white Gaussian noise. The binary stimulus was constructed by a choosing light intensity for each colour channel randomly between two values. In this case, since the linear filter may not be proportional to the STA [27], we extended Linear-Nonlinear model to a Generalized Linear Model (GLM). The results showed that the achromatic summed LN model performed poorly at high red contrast (∼0.2 correlation coefficient, Figure 5B) and there was a much broader gap in performance between the achromatic summed LN model and the CR-LN model. This is due to the fact that salamander retinal ganglion cells are much more sensitive to the blue colour than to the red colour (Discussion, Why is there a blue bias?). Therefore, at high blue contrast red-blue stimuli "appear" as achromatic blue stimuli for the ganglion cell. On the other hand at high red contrast the red-blue stimuli "appear" as chromatic to the ganglion cell because of the skew of the ganglion cell sensitivity to the blue spectra. As a result, at high red contrast achromatic model fails to capture the chromatic properties of red-blue stimuli comparing to CR-LN model. This result indicates that the achromatic summed LN model fails to describe colour stimuli encoding for both colour contrast conditions.

Finally, we also analysed the performance limits of our CR-LN model by constructing a full 2D-fit LN model (Figure 3C, S6). This model was constructed by calculating the filters for each of the two colour channels separately and then fitting a general two-dimensional nonlinearity. Since we did not observe all possible combinations of the generator signals we interpolated the two dimensional nonlinearity to obtain a smooth representation of the functional relation between the generator signals and firing rates. This model has minimal assumptions, but requires smoothing of the observed data. We found that the 2D-fit LN model performance was not significantly better than the CR-LN model (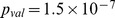
and 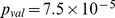 for high red and high blue contrasts respectively, Wilcoxon test, Table 1). Thus, the CR-LN model is sufficient to capture the basic features of the neuronal response.

### Retinal colour contrast adaptation can be represented as a rotation of colour space

**Figure 6:**
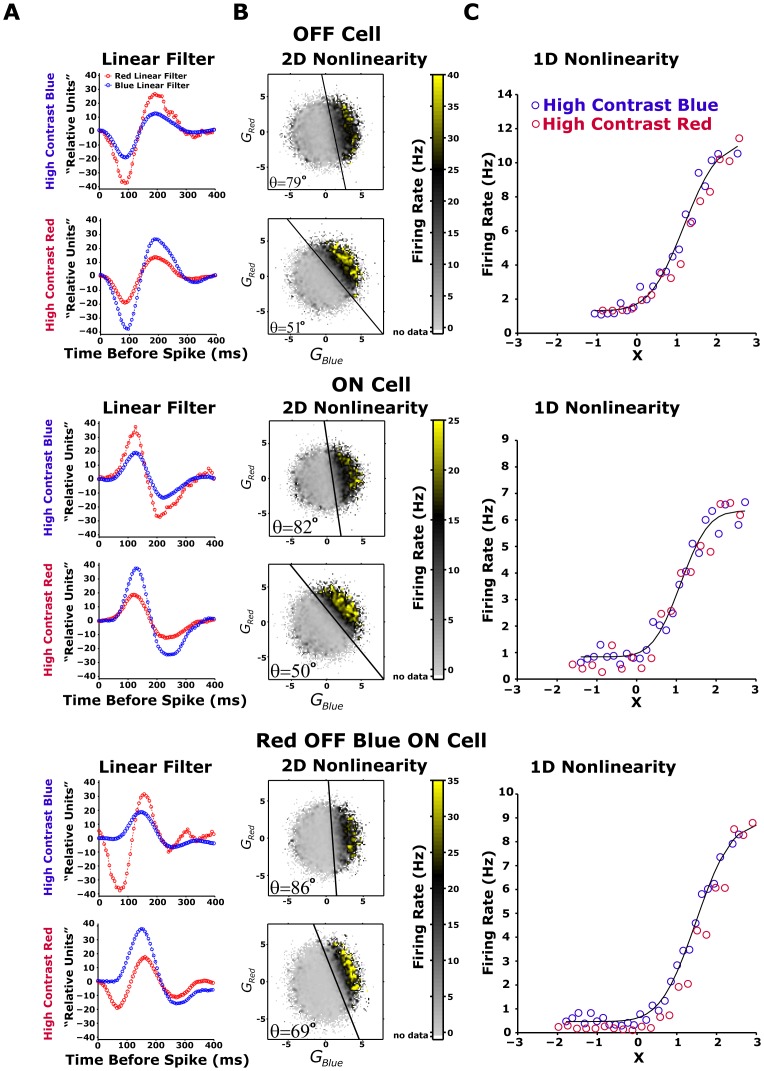
LN model of different ganglion cells in different colour contrasts. (A-C) LN model of ganglion cell firing after adaptation to colour contrast for red OFF blue OFF (upper panels), red ON blue ON (middle panels), and red OFF blue ON (lower panels) cells. The contrast modulation in this experiment was as described in Figure 2A, 2B: the high and low colour contrasts were 24% and 12% respectively. For LN model analysis, the last 50 s of the 100-s contrast presentations were used. (**A**) Scaled linear filter. Filter amplitude is higher for low contrast colours. Adaptive rescaling of each individual colour channel served to increase the gain of low contrast colour. (B) 2D nonlinearity at high red (lower panel) and high blue (upper panel) colour contrasts. Firing rate is shown as a function of both red and blue generator signals (red/blue colour stimulus weighted by red/blue linear filter), averaged during the experiment. The black line emphasizes the rotation angle 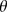 of 2D nonlinearity in the red-blue colour space. The angle 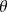 is modified with colour contrast modulation. (C) We replace the 2D nonlinearity with 1D nonlinearity, which is a function of 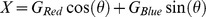. The nonlinearities for the red dominated (red curve) and blue dominated (blue curve) contrast modulations superimpose, suggesting that the difference between LN models between the colour contrast conditions was expressed solely in linear filter amplitude and in angle 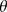 parameters.

Since the LN model we built is based on rotating the colour space in different conditions, we dubbed it the Chromatic Rotation LN (CR-LN) model. Using the CR-LN model (Materials and Methods, Equation 4), we explored the ways in which ganglion cells combine their spectral inputs, and found three different classes of cells. The first cell type is a Red-OFF/Blue-OFF intensity detector ganglion cell (Figure 6A, top panel). The second is the functional opposite of the first type, namely a Red-ON/Blue-ON cell (Figure 6A, middle panel). The third is a Red-OFF/Blue-ON cell that is sensitive to the difference between the red and blue channels (Figure 6A, bottom panel). The majority (∼90%) of the analysed ganglion cells were Red-OFF/Blue-OFF cells. The opponent Red-OFF/Blue-ON cells were very rare: we found only four cells out of the 399 ganglion cells whose functional types were analysed.

After identifying the three cell classes, we examined how the model parameters changed after adaptation to new colour stimuli. In mathematical terms, the adaptation of the cells to the new conditions could be expressed by changes in either the linear filters or the structure of the nonlinearity (or both). Since in constructing the model, the linear filter amplitude was determined by the requirement that the filter output have a unit variance, the only degree of freedom that could represent the adaptation was therefore the exact shape of the filter waveform. Here, we assumed that the filters are identical up to this scale factor and used this assumption in model validation experiments (Figure 2C). Thus, since the nonlinearity slope was not modified by colour contrasts (Figure 6C), the adaptation to colour contrasts manifests itself as changes in the nonlinearity by rotation of the colour space, described by rotation angle 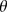. We then asked how this rotation angle 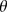, which determines the contribution of each colour channel to the output of the ganglion cell, changed according to different colour contrasts.

**Figure 7:**
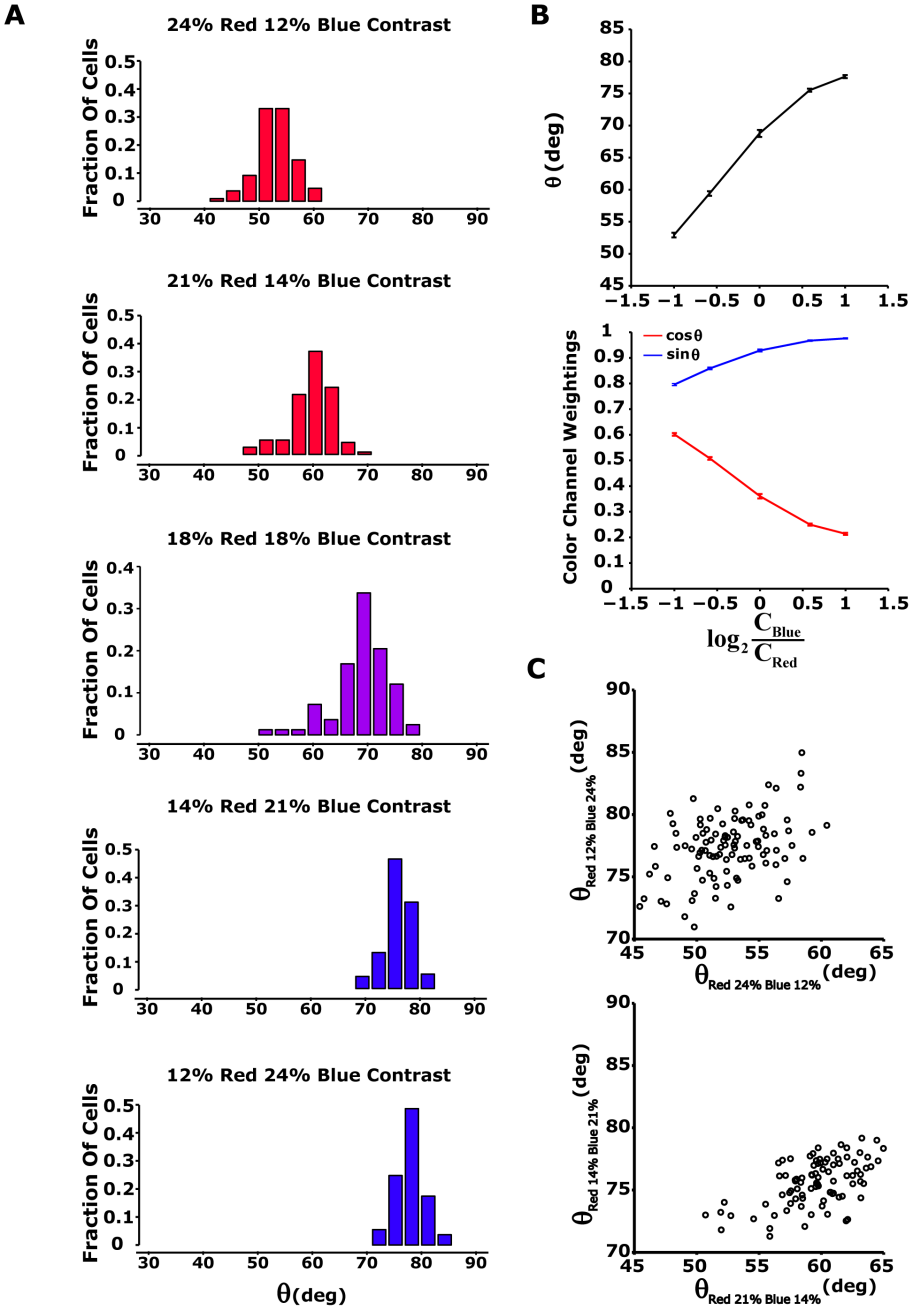
Modification of rotation angle 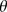 by colour contrast modulation. (A) Histogram of rotational angle for different colour contrast modulations. Upper and lower panels: rotation angle histogram (n = 117 cells, from one of four experiments) for 24% red and 12% blue contrast stimuli (red bars) and for 12% red and 24% blue contrast stimuli (blue bars). Second and forth panels: high colour contrast is lowered to 21%, while low colour contrast is changed to 14% (n = 109). Middle panel: 83 ganglion cells are excited by red blue stimuli with equal colour contrasts (18%). (B) Upper panel: summary of results from Figure 4A. The angle 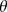 was averaged along the ganglion cell population as a function of colour contrast modulation: 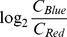 (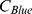 - contrast blue, 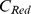 - contrast red). Error bars show standard error. Lower panel: average 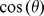 (red) and 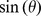 (blue). Here, 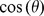 and 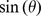 represent red and blue colour channel weightings, respectively, since 1D nonlinearity is a function of 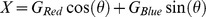. Error bars represent standard error. (C) Rotation angle 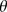 measured at high blue contrast plotted as a function of angle 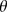 measured at high red contrast for all analyzed cells for 24% and 12% contrast (upper panel) and for 21% and 14% contrast (lower panel). It appears that there is a positive correlation between 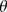 measured at high blue contrast and 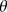 measured at high red contrast.

We found that all the analysed cells adapted to changes in colour contrast by changing the relative weights with which they combined the colour channels. This is equivalent to rotating the nonlinear function in the space of colour inputs. Such a change cannot be obtained by simply adjusting the gains of each of the colour channels independently, i.e., by contractions of the single channel axes, because rotation is a fundamental transformation of space that cannot be represented by a consecutive application of contractions and reflections. This change was apparent in all three classes of cells, and it matched the new balance between the contrasts of the different colour channels of the input stimuli (Figure 7B).

We estimated the rotation angle 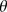 for each of the adapting cells we recorded, under several different colour contrast combinations, and found a clear dependency of the average angle 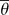 in the population on the red and blue contrasts (Figure 7A and see Table 2). In particular, the rotation in colour space of the weights assigned to the colour channels depended on the red-blue stimuli contrast ratio: when going from a *high red/low blue* stimulus contrast to a *low red/high blue* contrast, the red colour channel weight, 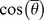, decreased, and the blue colour channel weight, 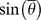, increased (Figure 7B). We also found that the angle in different conditions across the entire cell population behaved similarly (Figure 7C) with a weak correlation between the angles in different conditions.

We also constructed similar LN models for the photometric unit stimulus and found similar behaviour (Table 2). In addition, we examined the LN model with correlated red-blue stimuli (data not shown) and found that the rotation angles were similar to that found with uncorrelated red-blue stimuli (Table 2). To conclude, the results hold under different stimulus conditions.

### The rotation of the red-blue stimulus does not impose a rotation in ganglion cell response

The red-blue stimulus was designed to preserve the total contrast 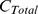 (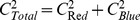, where 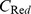 is red channel contrast and 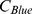 is a blue channel contrast) for both high-red/low-blue and low-blue/high-red stimulus conditions. This constraint imposes a relation between the red and blue channels in the stimulus that can be interpreted as a rotational constraint in stimulus space. This rotation in stimulus space may theoretically impose a rotation in response: if we define 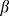 as the constant bias toward the blue colour channel (see Equation 11), and if the response 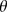 is only sensitive to the ratio of contrast values of the channels, then 
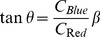
(11)and 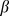 will not change between different contrast conditions.; otherwise there is an adaptation that is not described solely by the rotation in the stimulus. In order to test this hypothesis we calculated 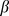 according to: 
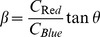
 (see Table 3). The results show that for contrasts between 24-12, 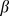 is proportional to 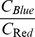 ratio; however, for higher contrasts ratios, the 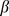 value changes significantly (see Table 3). This indicates that 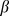 depends on contrast conditions and is not constant, or in other words, rotation in response is not driven solely by the rotation of the stimulus.

**Table 3:** The rotation in response is not driven solely by the rotation in red-blue stimulus.

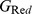	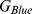	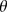	N	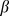
24	12	52±3.98	106	2.56±0.14
21	14	60±3.01	90	2.60±0.08
18	18	69±4.99	83	2.60±0.09
14	21	76±1.88	90	2.67±0.02
12	24	77±2.59	106	2.16±0.02
60	12	42±6.32	68	4.50±0.55
60	30	65±5.04	54	4.28±0.18
30	60	81±4.95	54	3.14±0.04
12	60	88±6.03	68	5.60±0.02

We have performed the experiments with different light statistics and estimated the average rotation angle 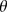 and 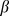 ± standard deviation, according to Equation 13 of the CR-LN model, over the population. N stands for number of analyzed ganglion cells.

### Natural colour scene statistics and retinal colour contrast adaptation

**Figure 8:**
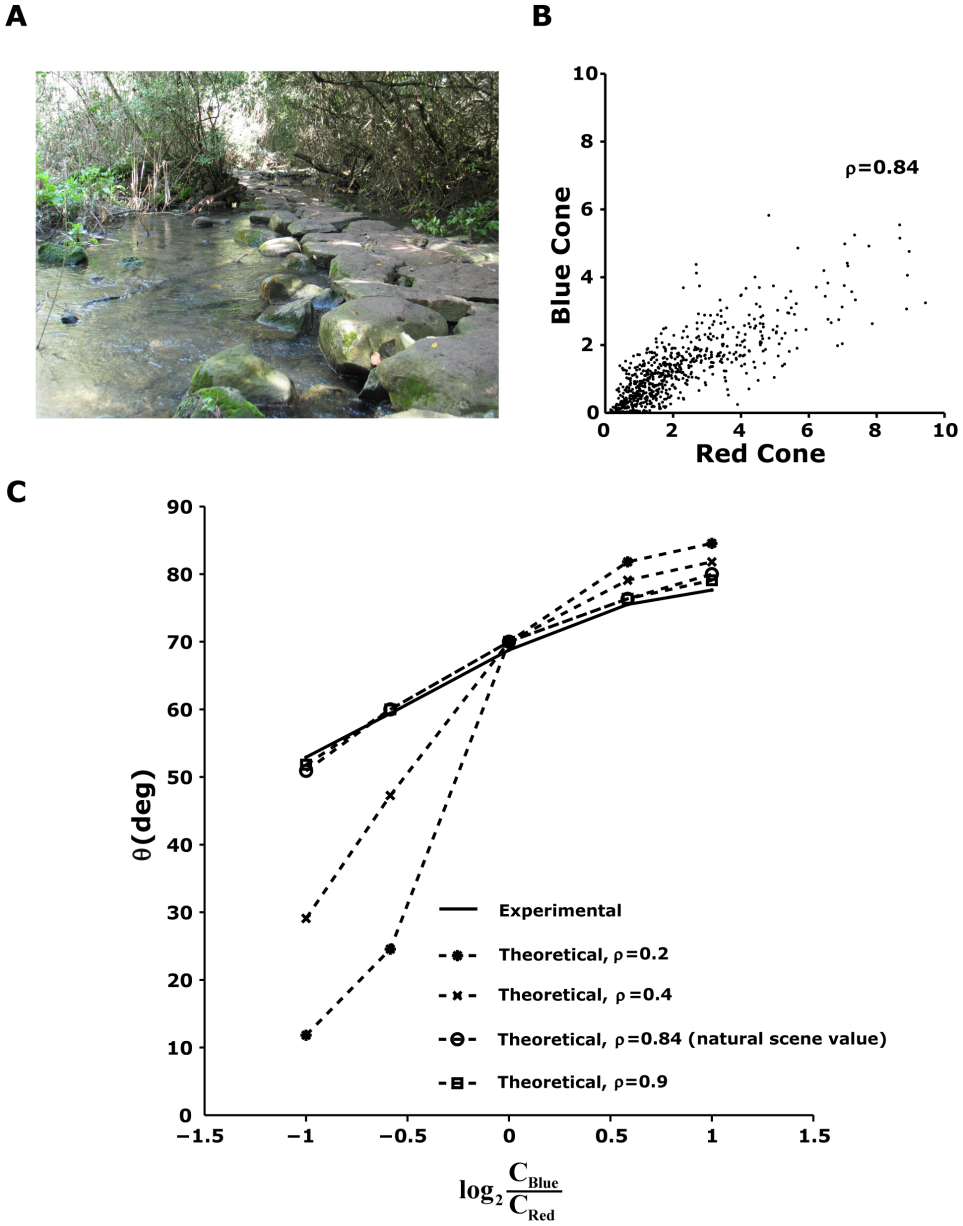
Natural scene statistics in the salamander natural environment can explain the selection of the adaptation parameter 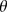. (A) A typical scene in the Tel Dan Natural Reserve (courtesy of Dr. Hava Goldstein). (B) To evaluate the correlation between light levels in the red and blue regions of the spectrum, each measured natural spectrum was convolved with the salamander red and blue photopigment absorption spectra to give us a measure of the amount of input available for the retinal circuitry in the two colours. The red and blue channels were shown to be strongly related, with a correlation coefficient of 0.84. (C) For each ratio between the contrasts in the two input channels, we found the optimal angle 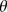 that maximizes the information transfer between the input and the response. We found that if the two input variables were strongly correlated, the value of the experimentally measured angle from the retina coincides with the value we obtained from the model given the bias in the natural environment toward the blue channel. This suggests that the retinal circuitry optimally selects the adaptation parameter to maximize information transmission.

Why does the colour channel weighting of the ganglion cells adapt to the different combinations of colour contrasts the way it does? We hypothesized that the adaptive response properties of the ganglion cells were related to the spectral statistics of natural scenes. We therefore measured 720 light spectra in a salamander natural habitat at the Tel Dan Nature Reserve in northern Israel (see Materials and Methods) [29]. This reserve has characteristics similar to those in the natural habitat of the tiger salamander, which spends most of the year underground, emerging from its burrow only during the breeding season [33]. At that time of year, tiger salamanders can be seen in the daytime in the proximity of water (breeding ponds), but only under dense vegetation, as they can rarely be found in direct sunlight (see, for example, Figure 8A). To understand the broad-band spectra stimuli that we measured in the natural scene in terms of the inputs to the red and blue photoreceptors, we projected the measured spectral intensities on the blue and red photoreceptor absorption spectra of the tiger salamander (Materials and Methods, Equation 7). We found that the equivalent red and blue channels were strongly correlated in the natural environment, with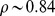 (Figure 8B). This strong correlation seems to be a general feature of natural scenes as reported by several other studies [34]–[37]. The contrast of the red channel was found to be ∼0.88 over the entire dataset, and the contrast of the blue channel was ∼0.73.

We then asked how optimal encoding of the natural scene colour statistics would be reflected by the parameters of a CR-LN model. We approximated the ganglion cell's inputs using two random variables, the red input 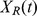 and the blue input 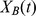, which were normally distributed with standard deviations and a correlation coefficient given by 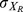, 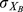, and 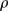, respectively. In this case the Chromatic Rotation LN model can be written as:
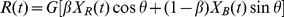
(12)where 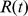 is the retinal output, 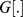 is a static nonlinearity, 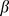 is the constant bias toward one of the channels (blue in our case), and 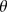 is the adaptation parameter when the retina encounters different environments. We choose the optimal theta 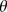 by maximizing the mutual information between the input and response, which is:



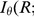
(13)If we ignore neural response noise, or focus only on the firing rates, then this implies that 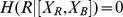. We then find the optimal 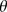 by maximizing the entropy of the response 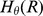 from Equation 13, for the same stimulus mean intensity and standard deviation that were used in the experiments, under different 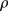 values. This model has one free parameter, 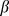 (Equation 12), which determines the balance between the input blue and the red channels, which we can infer from the case 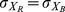. Note that for all measured OFF (Figure 7B, upper panel) and ON (data not shown) ganglion cells, the responses were biased towards the blue channel contrast with an average 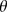 ∼70 degrees.

We found experimentally that the strong colour correlations in natural scenes were reflected in the nature of retinal adaptation to different colour statistics (Figure 8C). Analysis of the optimal 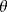 for different ratios of the contrasts of the red and blue channels showed that if there was no correlation between the contrasts of the colour channels, then the optimal 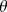 was constant and the retinal response was completely dominated by the channel with the maximal contrast (Equation 12). For correlation coefficient values that were much smaller than the values observed in natural scenes, the optimal 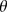 was far from that found experimentally (Figure 8C). Finally, we found that the experimental and theoretical curves were very similar when we took into account the strong correlations between the two channels (i.e., 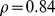), which suggests that the angle 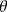 may be optimally selected by the retinal circuitry to match the high correlation between colour channels found in natural environments.

## Discussion

We found that colour contrast adaptation begins in the retina, where adaptation to colour can be understood in terms of two working components. The *first component*, contrast gain control, works independently in different colour channels by increasing ganglion cell sensitivity at low contrast and decreasing sensitivity at high contrast. The increase in cell sensitivity expanded the signal-to-noise ratio of ganglion cell responses to the low contrast channel. Likewise, the reduction in gain helps prevent saturation of the ganglion cell response in high contrast channels. This component of colour contrast adaptation is also common to the adaptation processes for achromatic stimuli. The *second component* controls the two-dimensional nonlinearity function that translates the output of the linear filters in each of the colour channels to the firing rates of the ganglion cell. We showed that rotation of the colour space changes the basis of the input colour space to the new one that has two orthogonal directions: one changes the firing rate and the second degenerates, i.e., it has no effect on the ganglion cell response. As a result, colour contrast adaptation can be described as a function of a single angle that weights the input from the two colour channels. This adaptation is the equivalent of rotating the colour space representation, an operation that cannot be explained by a simple contraction of the axes.

The common view of the encoding of colour information by the retina to the brain is that of merging of the colour-tuned photoreceptor output into colour-opponent ganglion cells [2], [3], [5], [38]. Our results suggest that ganglion cells do not have a static colour identity, but rather than the chromatic preference changes with the stimuli.

Since photoreceptors lack the ability to adapt to contrast [14], the colour adaptation we demonstrated here should be attributed to the ability of the retinal network to adapt to the spectral properties of the environment. Our study of the spectra the salamander encounters in its natural habitat showed that there is a strong correlation between the blue (short) and red (long) wavelengths. Using a model of information encoding by the two colour channels, we showed that the retina seemed to respond to colour stimuli such that it maximized the information transmitted by ganglion cells about the spectral content of natural scenes.

### Relation of current work to studies on colour vision in humans and primates

Human colour perception is based on three fundamental sensory receptors: red (L – long wavelength sensitive), green (M – middle wavelength) and blue (S – short wavelength), which the retina then processes in a colour-opponent fashion [5]. This colour opponent processing serves to remove the correlation that is present between pure chromatic channels due to the overlapping spectral sensitivities of the L, M and S cone photoreceptors [4]. In the current work only 1% of the tiger salamander retinal ganglion cells were found to be “colour opponent” cells. It is possible that since there is less than 5% overlap between long and short salamander cones, the de-correlation by colour opponent mechanism may be less important in the tiger salamander than in humans or in Old World primates. Moreover, the identified retinal colour opponent ganglion cells adapt to chromatic contrast modulation in a manner similar to non- colour opponent cells.

The evidence for chromatic contrast adaptation first came from psychophysical experiments that identified the adaptation of colour perception along L-M and S-(L+M) colour opponent channels following chromatic modulation [39], [40]. Later electrophysiological experiments in primates revealed the colour opponent channels in LGN [41]. However, these cells were found to have little susceptibility to colour adaptation, which thus hinted that higher visual areas could be origin for colour contrast adaptation. In fact, many studies in primates have reported evidence of chromatic adaptation in V1 [42]–[44]. Here we found that in the tiger salamander, colour contrast adaptation starts in the retina. As there is no current evidence negating its existence – it is possible that colour contrast adaptation will also be found in the primate retina.

### Rod contribution to colour contrast adaptation

In our experiment we have not distinguished between the contribution of either rods or cones to colour contrast adaptation. There are two types of rods in salamander retina: short wavelength sensitive rod with peak sensitivity at ∼420 nm and a medium wavelength sensitive rod with peak sensitivity at ∼510 nm [45]. This indicates that salamander rod vision is skewed to the lower wavelength spectra. Moreover, the rods are not fully saturated at the range of light intensities used in the experiment [46].

In the current study we investigated the retinal contrast adaptation to spectral properties of the environment, therefore the proposed colour contrast adaptation model is independent of retinal spectral sensitivity properties. However, this is not the case for the photometric stimuli, where the rod stimulation will produce a higher retinal excitation for the blue spectra. This asymmetry in retinal spectral sensitivity due to the rod input can explain the high sensitivity of salamander retinal ganglion cells to the blue spectra of visual stimuli (see next paragraph: Why is there a blue bias?). The results of our natural scene statistics analysis are not influenced by the specific input pathway (rod or cone), since the ganglion cell spectral sensitivity is incorporated into the encoding model, and not dependent on a specific mechanism.

One should keep in mind that the issue of rod contribution is important on the mechanistic level. However, our analysis is not a mechanistic analysis of the retina adaptation to colour contrast but rather a phenomenological assessment of the adaptation process. In previous efforts to understand other types of retinal contrast adaptation, the first step was characterization of the process on the retinal output level and only later the discussion of the mechanism took place. Our work provides the first step and leaves the mechanism for future studies.

In order to test the rod contribution to colour contrast adaptation it is possible to design an experiment that eliminates the rod output to the retinal circuitry by the use of high intensity stimuli saturating the rods, but not the cones. Such an experiment will require a specific stimulation equipment with higher intensity range then a computer monitor, which was used in the current study.

### Why is there a blue bias?

The investigation of the CR-LN model under equal contrast conditions revealed that the salamander retina has an inherent bias toward the blue colour contrast.

One explanation is that this bias is a result of the higher sensory input to the blue channel due to the rods stimulation, since tiger salamanders have rods with peak sensitivity in the short and medium wavelength [45].

Another explanation is that this high sensitivity to the blue spectra is an inherited property of the salamander retinal circuitry, for example, due to the facilitation of the blue channel during sensory processing. Such a property can assist salamanders to thwart avian predators. The avian predator will then generate a clear signal over the background of the blue sky. Additional support for this explanation can be found in the anatomical observation that the salamander's eyes are situated on the top of its head, pointing upward.

### Limitations of our analysis of natural environment colour encoding

Our analysis of natural scene statistics and its relation to the colour contrast adaptation at the level of the ganglion cells is limited in scope, as we used only a single feature of the natural scene; namely, the high correlations between the two colour channels. In nature, the colour channels do not vary around a single mean but rather the mean itself varies during the day. In addition, in our analysis of natural scenes we omitted dynamical properties of the stimulus.

### Differences between laboratory and natural environment stimuli

We used a CRT monitor in our experiments to present the stimulus to the salamander retina. While the monitor is the standard vehicle for presenting visual stimuli in many electrophysiological and behavioural studies, its limited dynamic range restricted our ability to perform experiments at wider light intensity ranges. However, despite the experimental limitations on light intensity and contrast (24% contrast in the laboratory compared to 150-400% in the natural environment), the salamander retina showed significant remodelling of the colour coding scheme in response to colour contrast modulation. We believe that extending the ranges of light intensity and stimuli contrast would result in more significant adaptation, since the visual system follows some form of a Weber law adjusted to colour contrast [47].

### The salamander retina has additional colour channels in the UV region

The tiger salamander (*Ambystoma tigrinimum*) has red, blue, and UV cones [25], [48]. In this study, we examined colour contrast adaptations to red and blue colour modulations and ignored salamander colour vision in the UV light spectrum. Two technical issues of our experimental setup dictated that we neglect the UV light spectrum in our investigation of adaptive colour coding. First, the addition of a third colour dimension would significantly complicate the analysis of the salamander ganglion colour coding scheme. Second, the use of UV visual stimuli is technically complex, as it would have required replacing the CRT monitor with a UV light source. The main objective of the current work was to demonstrate and describe, as simply as possible, how colour contrast modulation modifies the retinal colour coding scheme. Although the red and blue colour channels were well suited to this purpose, our exclusive use of these colour channels neglected the potential contribution of the salamander's tri-chromatic cone colour vision, and therefore, additional adaptation mechanisms may exist.

### Interpretation of the CR-LN model in terms of retinal circuitry and function

The LN model is a phenomenological model with only a loose connection to concrete neuronal elements such as biopolar or amacrine cells. Thus, we cannot draw concrete conclusions from the model regarding where the colour signals are integrated. However, there is evidence that the achromatic contrast adaptation results from combined activity of bipolar and ganglion cells [12], [14], [20], [49] which suggests that a similar combination is responsible for colour contrast adaptation.

## Supporting Information

Figure S1
**Calibration of the CRT monitor spectrum.** (**A**) The spectral output of CRT monitor for a screen command value was at 255 RGB (see Methods for details about spectrum measurements). The red and blue monitor electron gun spectral peaks (solid curves; peak wavelength in nm, adapted from Makino et al.) coincide closely with tiger salamander cone peak sensitivities (dashed curves with circles) Rod spectral sensitivities appear in dashed curves. (B) For each screen command value in the range of 5 to 255 RGB, we calculated the total intensity output of the monitor by taking the integral over the wavelengths of the blue and red monitor guns. In the experiments, we used light intensity range between 0 and 0.34 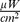.(TIF)

Figure S2 **Spectral contrast adaptation in salamander retinal ganglion cells using photometrically calibrated stimuli.** (A) An example of the red-blue stimulus, photometrically calibrated (see Methods), used in the experiment; the mean intensity of each color and the total light intensity were held constant. (B) Schematic representation of the color contrast modulation used in the experiment. Each 200-s segment of the red-blue stimulus contained 100 s of random flicker at high red (24% contrast) and low blue contrast (12% contrast) followed by 100 s at low red and high blue contrast. Total light contrast remained constant throughout the experiment. (C) Raster plot and peri-stimulus time histogram (PSTH) in response to 52 contrast modulation cycles, calculated with 4-s bins for OFF (upper panel) and ON (lower panel) ganglion cells. The PSTH curves were fitted with exponential curves (green) with time constants in the range of 5–20 s.
(TIF)

Figure S3
**LN model of different ganglion cells in different color contrasts using photometrically calibrated stimuli.** (A-C) LN model of ganglion cell response after adaptation to color contrast, for a red OFF-blue OFF cell (upper panels) and a red ON-blue ON (middle panels). The contrast modulation in this experiment was as described in Figure S2A, S2B: the high and low color contrasts were 24% and 12% respectively. Analysis of the LN relied on the last 50 s of the 100-s contrast presentations. (A) Linear filter amplitude is higher for low contrast colors. Adaptive rescaling of each individual color channel increased the gain of low contrast color. (B) 2D nonlinearity for high red (lower panel) and high blue (upper panel) color contrasts. Firing rate is shown as a function of both red and blue generator signals (red/blue color stimulus weighted by red/blue linear filter), averaged during the experiment. The black line emphasizes the rotation angle of 2D nonlinearity in the red-blue color space. The angle changes with color contrast modulation. (C) We replaced the 2D nonlinearity with 1D nonlinearity, which is a function of 
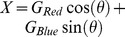
. The nonlinearities for the red dominated (red curve) and blue dominated (blue curve) contrast modulations are superimposed, reflecting that the difference between LN models between the color contrast conditions manifested itself solely in the amplitude of the linear filter and in angle parameters.(TIF)

Figure S4 **Spectral contrast adaptation in salamander retinal ganglion cells using red-blue correlated stimuli.** (A) An example of the red-blue correlated stimulus (correlation coefficient ρ = 0.84, as was found in salamander's natural habitat, see Figure 8B) used in the experiment; the mean intensity of each color channel and the total light intensity were held constant. (B) Schematic representation of the color contrast modulation used in the experiment. Each 200-s segment of the red-blue stimulus trial contained 100 s of random flicker at high red (24% contrast) and low blue contrast (12% contrast) followed by 100 s at low red and high blue contrast. Total light contrast remained constant throughout the experiment. (C) Raster plot and peri-stimulus time histogram (PSTH) in response to 52 contrast modulation cycles, calculated with 4-s bins for OFF (upper panel) and ON (lower panel) ganglion cells. The PSTH curves were fitted with exponential curves (green) with time constants in the range of 5-20 s.
(TIF)

Figure S5
**LN model of different ganglion cells in different color contrasts using red-blue correlated stimuli.** (A-C) LN model of ganglion cell's response, after adaptation to color contrast for red OFF-blue OFF (upper panels) and red ON-blue ON (middle panels). The contrast modulation in this experiment was as described in Figure S4A, S4B: the high and low color contrasts were 24% and 12% respectively. For LN model analysis, the last 50 s of the 100-s contrast presentations were used. (A) Filter amplitude was higher for low contrast colors. Adaptive rescaling of each individual color channel served to increase the gain of low contrast color. (B) 2D nonlinearity at high red (lower panel) and high blue (upper panel) color contrasts. Firing rate is shown as a function of both red and blue generator signals (red/blue color stimulus weighted by red/blue linear filter), averaged during the experiment. The black line emphasizes the rotation angle of 2D nonlinearity in the red-blue color space. The angle is modified with color contrast modulation. (C) We replace the 2D nonlinearity with 1D nonlinearity, which is a function of 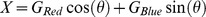. The nonlinearities for the red dominated (red curve) and blue dominated (blue curve) contrast modulations superimpose, suggesting that the difference between LN models between the color contrast conditions was expressed solely in linear filter amplitude and in angle parameters.(TIF)

Figure S6 **Performance of CR-LN model compared to general color-weight model at 60% high 12% low contrast stimuli.** Left column: high red (60%) and low blue (12%) color contrast. Right column: high blue (60%) and low red (12%) color contrast. (A) Intensities of red and blue light stimuli (red and blue curves) used in the experiment. (B) Achromatic summed LN model (Figure 3B) prediction compared to the output of the chromatic rotation LN model (Figure 3A), and to experimental data. Data was averaged over 20 trials (bin size 33 ms). The achromatic summed LN model output (grey line), constructed from a separate data set, fails to follow the data (green line) at high red contrast. (C) Correlation coefficient for the CR-LN model plotted as a function of correlation coefficient for the achromatic summed LN model. The correlation coefficient for the achromatic summed LN model is lower than the correlation coefficient for the CR-LN model for almost all tested cells (n = 68).
(TIF)
