# Supplementary figures and images for: Adaptive Colour Contrast Coding in the Salamander Retina Efficiently Matches Natural Scene Statistics
Source: PLoS One. 2013 Oct 30;8(10):e79163. doi: 10.1371/journal.pone.0079163 (PMC3813611; doi:10.1371/journal.pone.0079163)

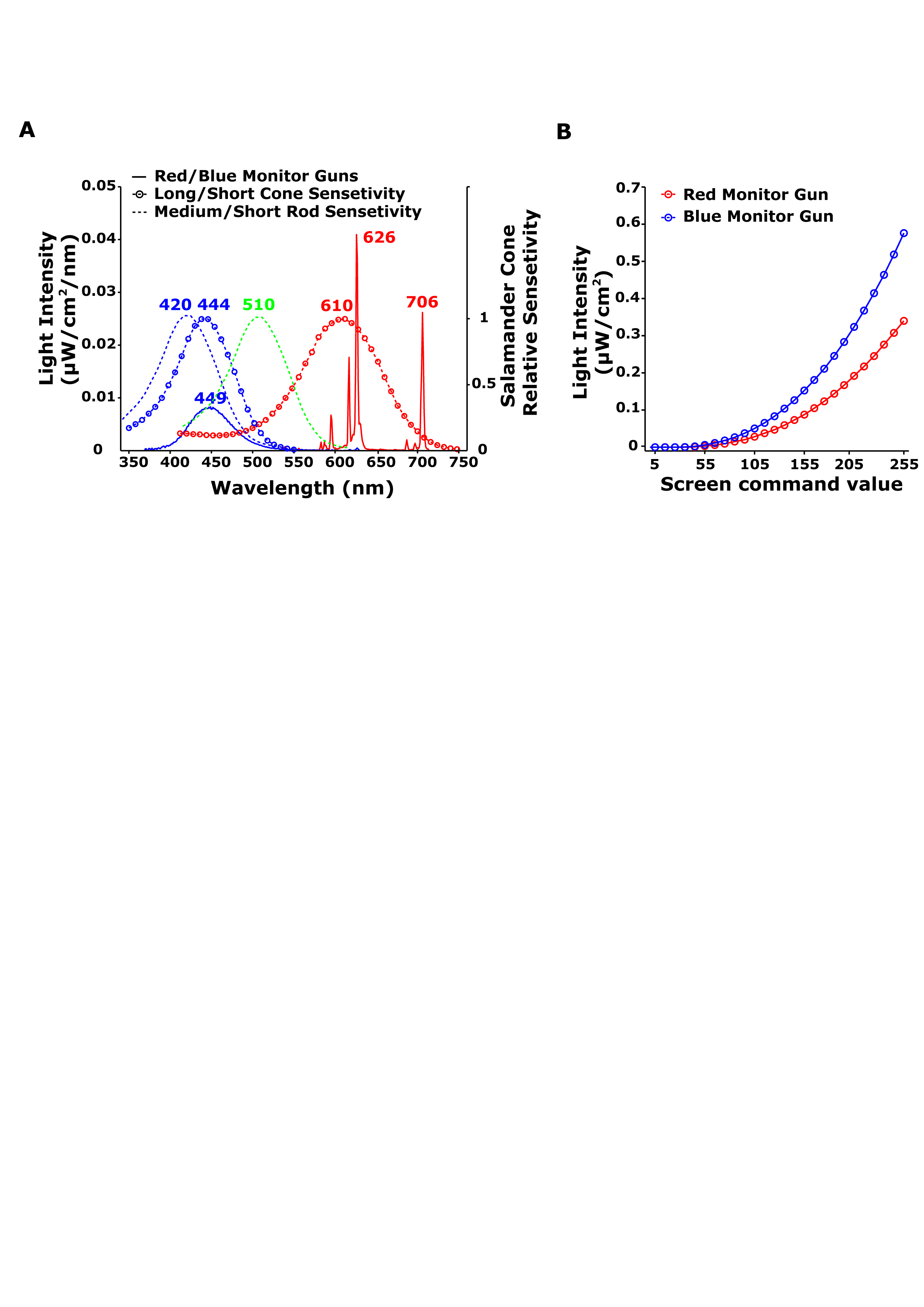

Supplement: Figure S1 — Calibration of the CRT monitor spectrum. (A) The spectral output of CRT monitor for a screen command value was at 255 RGB (see Methods for details about spectrum measurements). The red and blue monitor electron gun spectral peaks (solid curves; peak wavelength in nm, adapted from Makino et al.) coincide closely with tiger salamander cone peak sensitivities (dashed curves with circles) Rod spectral sensitivities appear in dashed curves. (B) For each screen command value in the range of 5 to 255 RGB, we calculated the total intensity output of the monitor by taking the integral over the wavelengths of the blue and red monitor guns. In the experiments, we used light intensity range between 0 and 0.34 . (TIF) [file pone.0079163.s001.tif]

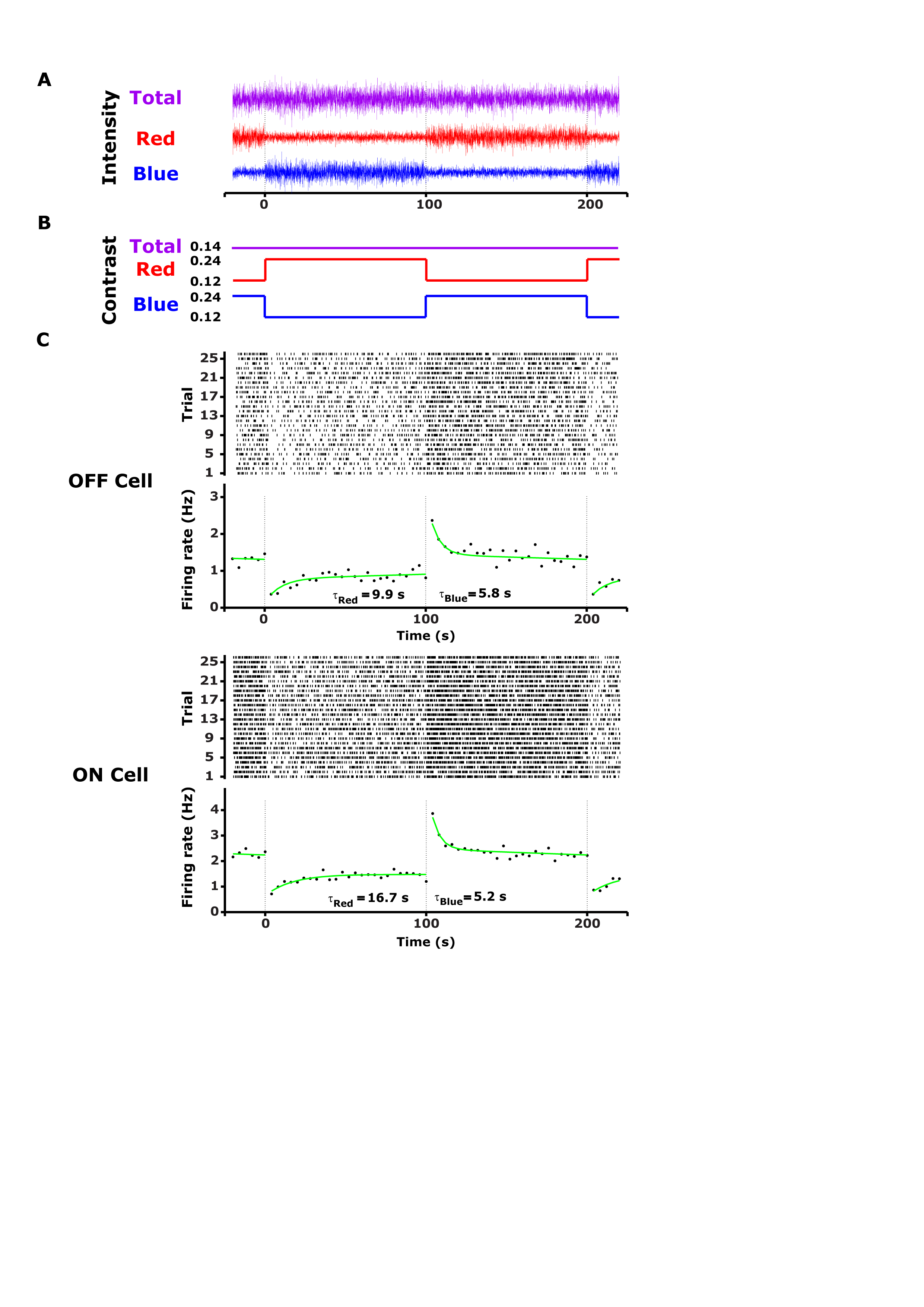

Supplement: Figure S2 — Spectral contrast adaptation in salamander retinal ganglion cells using photometrically calibrated stimuli. (A) An example of the red-blue stimulus, photometrically calibrated (see Methods), used in the experiment; the mean intensity of each color and the total light intensity were held constant. (B) Schematic representation of the color contrast modulation used in the experiment. Each 200-s segment of the red-blue stimulus contained 100 s of random flicker at high red (24% contrast) and low blue contrast (12% contrast) followed by 100 s at low red and high blue contrast. Total light contrast remained constant throughout the experiment. (C) Raster plot and peri-stimulus time histogram (PSTH) in response to 52 contrast modulation cycles, calculated with 4-s bins for OFF (upper panel) and ON (lower panel) ganglion cells. The PSTH curves were fitted with exponential curves (green) with time constants in the range of 5–20 s. (TIF) [file pone.0079163.s002.tif]

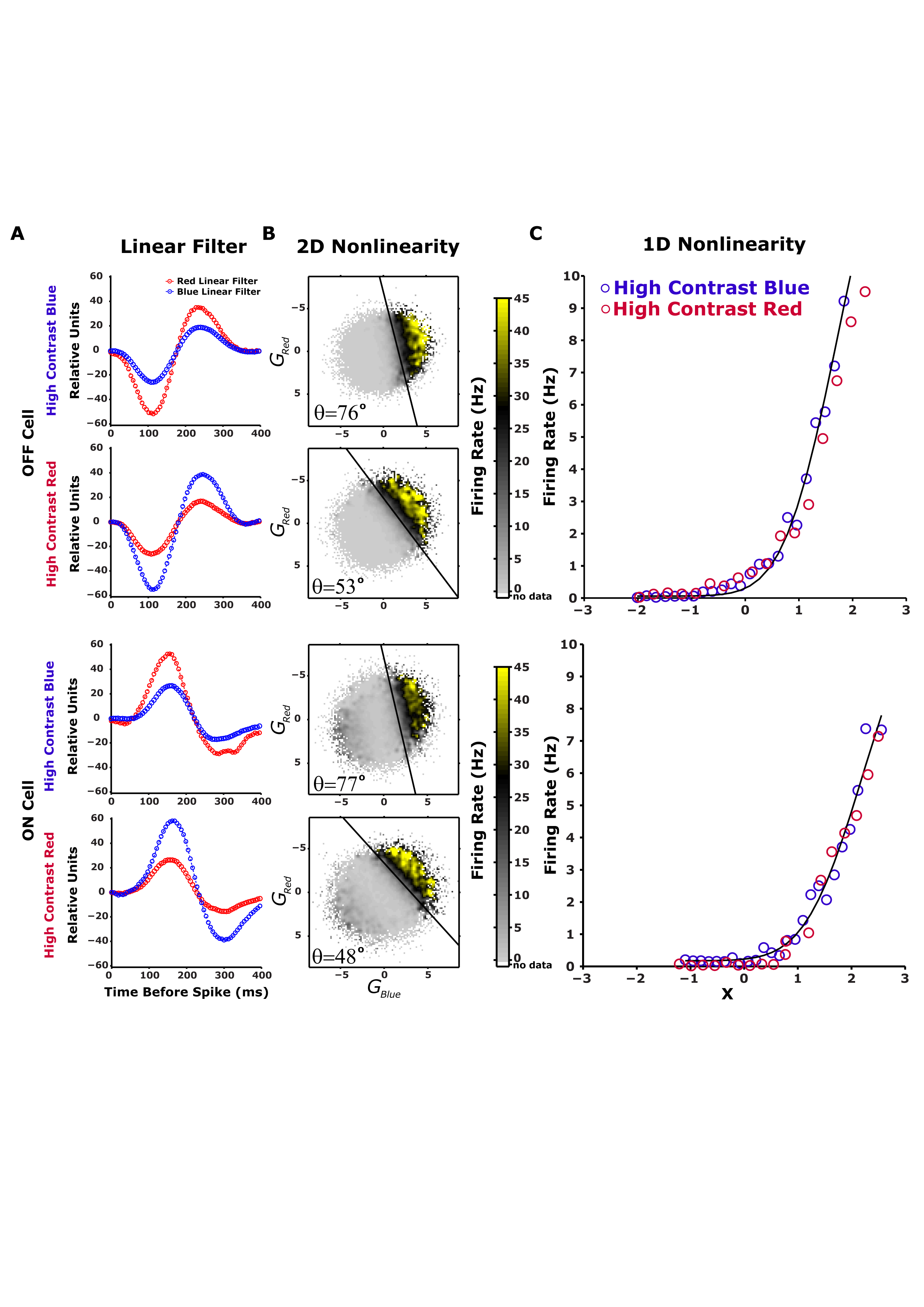

Supplement: Figure S3 — LN model of different ganglion cells in different color contrasts using photometrically calibrated stimuli. (A-C) LN model of ganglion cell response after adaptation to color contrast, for a red OFF-blue OFF cell (upper panels) and a red ON-blue ON (middle panels). The contrast modulation in this experiment was as described in Figure S2A, S2B: the high and low color contrasts were 24% and 12% respectively. Analysis of the LN relied on the last 50 s of the 100-s contrast presentations. (A) Linear filter amplitude is higher for low contrast colors. Adaptive rescaling of each individual color channel increased the gain of low contrast color. (B) 2D nonlinearity for high red (lower panel) and high blue (upper panel) color contrasts. Firing rate is shown as a function of both red and blue generator signals (red/blue color stimulus weighted by red/blue linear filter), averaged during the experiment. The black line emphasizes the rotation angle of 2D nonlinearity in the red-blue color space. The angle changes with color contrast modulation. (C) We replaced the 2D nonlinearity with 1D nonlinearity, which is a function of . The nonlinearities for the red dominated (red curve) and blue dominated (blue curve) contrast modulations are superimposed, reflecting that the difference between LN models between the color contrast conditions manifested itself solely in the amplitude of the linear filter and in angle parameters. (TIF) [file pone.0079163.s003.tif]

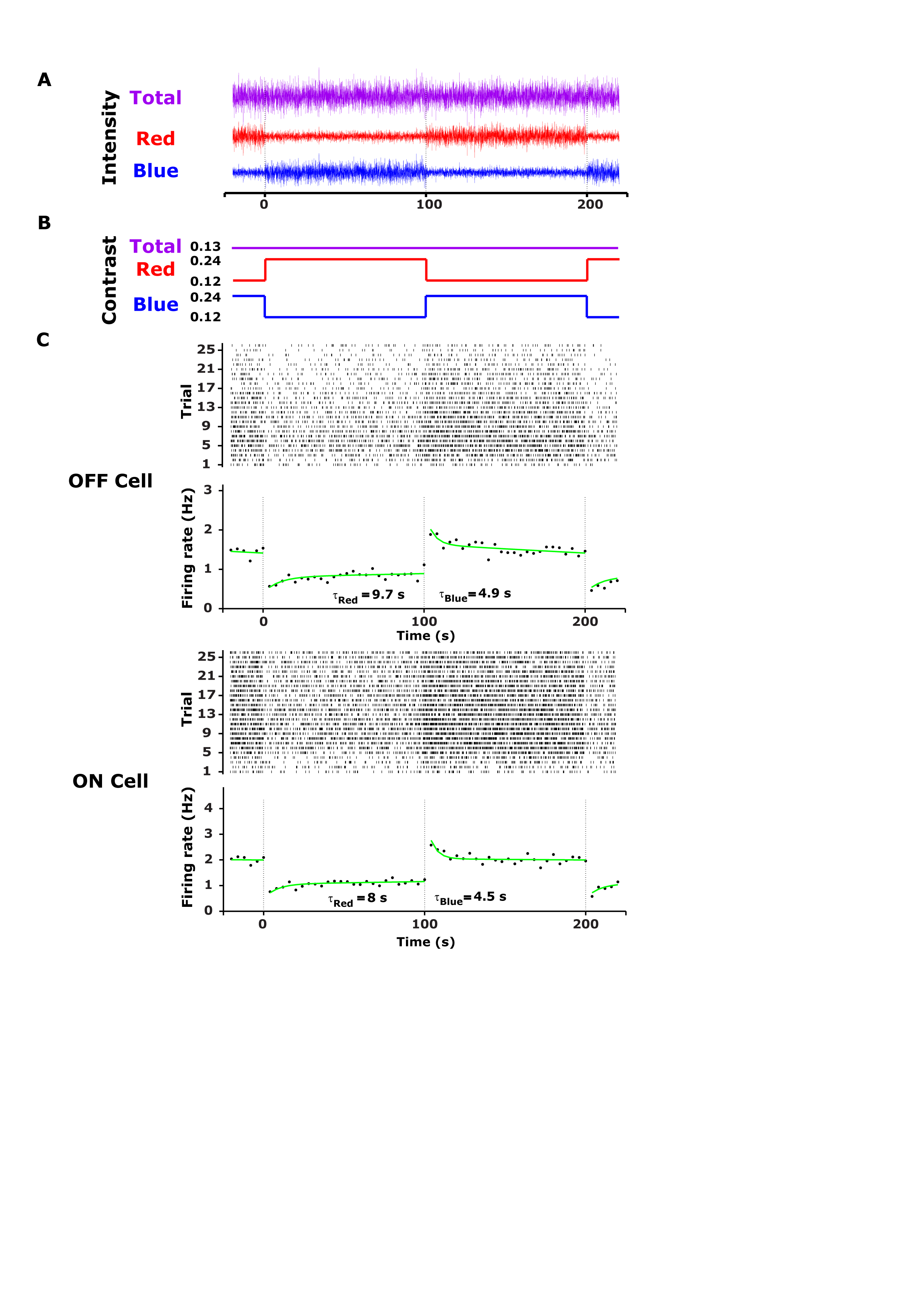

Supplement: Figure S4 — Spectral contrast adaptation in salamander retinal ganglion cells using red-blue correlated stimuli. (A) An example of the red-blue correlated stimulus (correlation coefficient ρ = 0.84, as was found in salamander's natural habitat, see Figure 8B) used in the experiment; the mean intensity of each color channel and the total light intensity were held constant. (B) Schematic representation of the color contrast modulation used in the experiment. Each 200-s segment of the red-blue stimulus trial contained 100 s of random flicker at high red (24% contrast) and low blue contrast (12% contrast) followed by 100 s at low red and high blue contrast. Total light contrast remained constant throughout the experiment. (C) Raster plot and peri-stimulus time histogram (PSTH) in response to 52 contrast modulation cycles, calculated with 4-s bins for OFF (upper panel) and ON (lower panel) ganglion cells. The PSTH curves were fitted with exponential curves (green) with time constants in the range of 5-20 s. (TIF) [file pone.0079163.s004.tif]

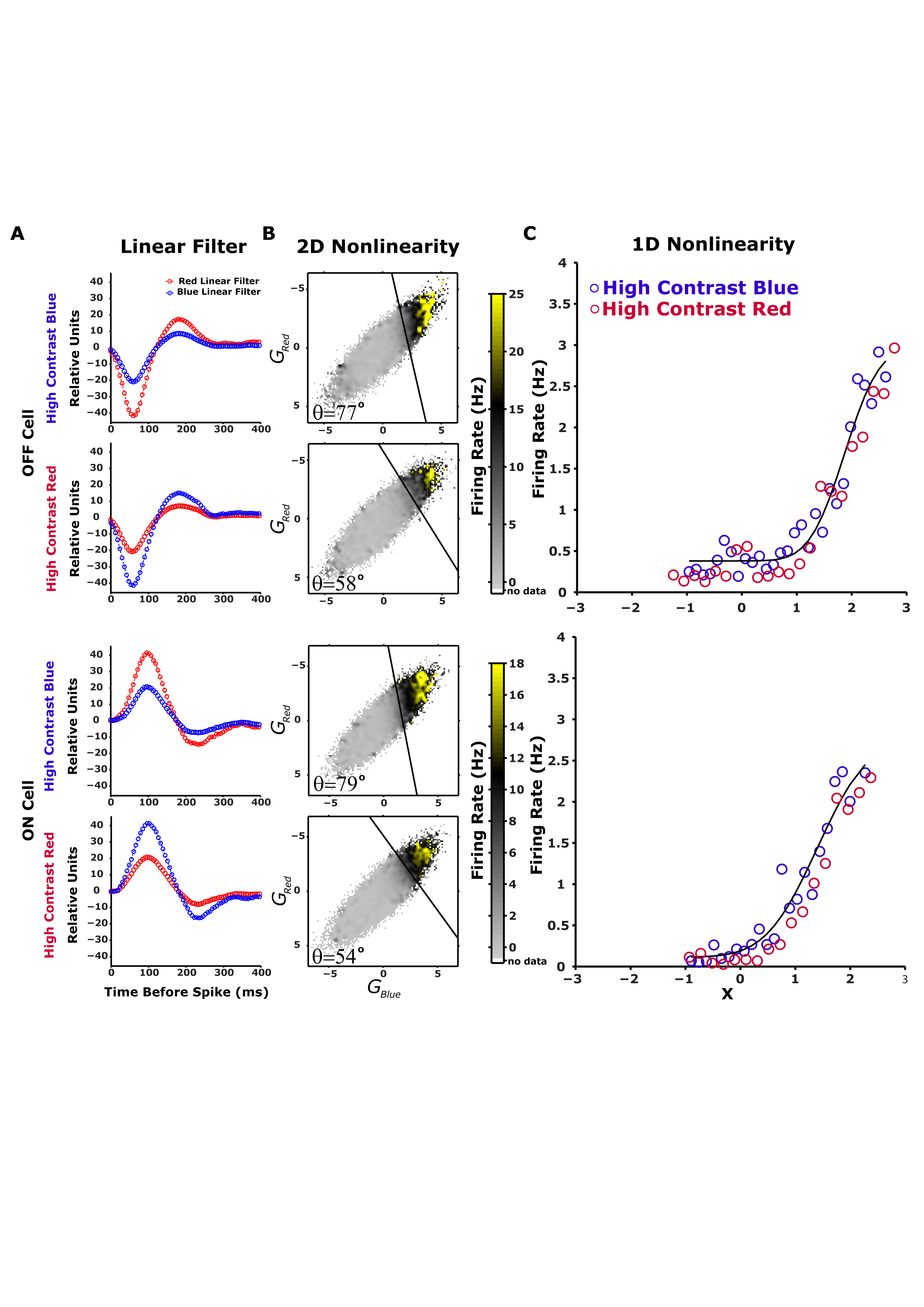

Supplement: Figure S5 — LN model of different ganglion cells in different color contrasts using red-blue correlated stimuli. (A-C) LN model of ganglion cell's response, after adaptation to color contrast for red OFF-blue OFF (upper panels) and red ON-blue ON (middle panels). The contrast modulation in this experiment was as described in Figure S4A, S4B: the high and low color contrasts were 24% and 12% respectively. For LN model analysis, the last 50 s of the 100-s contrast presentations were used. (A) Filter amplitude was higher for low contrast colors. Adaptive rescaling of each individual color channel served to increase the gain of low contrast color. (B) 2D nonlinearity at high red (lower panel) and high blue (upper panel) color contrasts. Firing rate is shown as a function of both red and blue generator signals (red/blue color stimulus weighted by red/blue linear filter), averaged during the experiment. The black line emphasizes the rotation angle of 2D nonlinearity in the red-blue color space. The angle is modified with color contrast modulation. (C) We replace the 2D nonlinearity with 1D nonlinearity, which is a function of . The nonlinearities for the red dominated (red curve) and blue dominated (blue curve) contrast modulations superimpose, suggesting that the difference between LN models between the color contrast conditions was expressed solely in linear filter amplitude and in angle parameters. (TIF) [file pone.0079163.s005.tif]

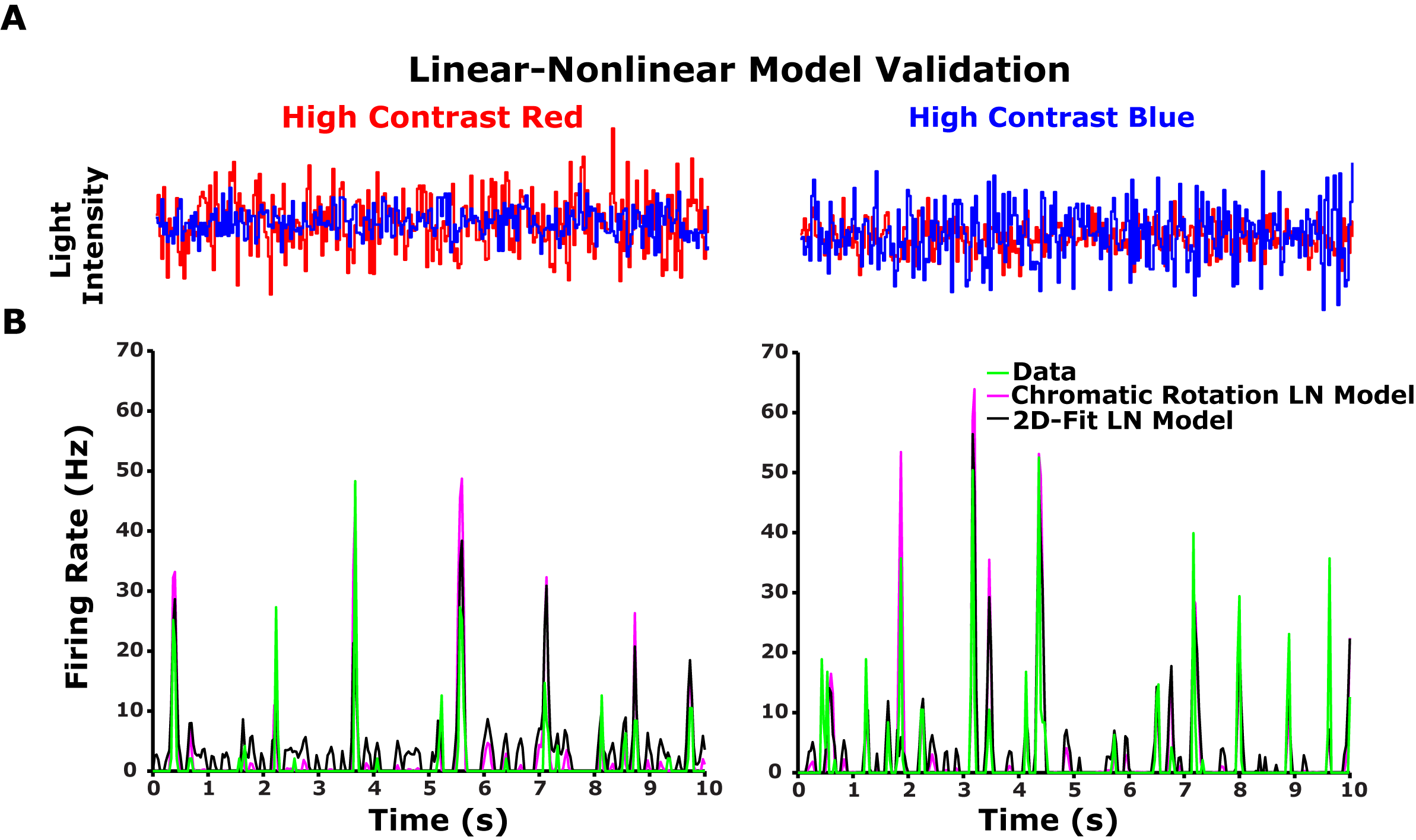

Supplement: Figure S6 — Performance of CR-LN model compared to general color-weight model at 60% high 12% low contrast stimuli. Left column: high red (60%) and low blue (12%) color contrast. Right column: high blue (60%) and low red (12%) color contrast. (A) Intensities of red and blue light stimuli (red and blue curves) used in the experiment. (B) Achromatic summed LN model (Figure 3B) prediction compared to the output of the chromatic rotation LN model (Figure 3A), and to experimental data. Data was averaged over 20 trials (bin size 33 ms). The achromatic summed LN model output (grey line), constructed from a separate data set, fails to follow the data (green line) at high red contrast. (C) Correlation coefficient for the CR-LN model plotted as a function of correlation coefficient for the achromatic summed LN model. The correlation coefficient for the achromatic summed LN model is lower than the correlation coefficient for the CR-LN model for almost all tested cells (n = 68). (TIF) [file pone.0079163.s006.tif]
